# Neurotoxic Metal Coexposures and Neurodevelopment

**DOI:** 10.1289/ehp.1205004

**Published:** 2012-06-01

**Authors:** José G. Dórea

**Affiliations:** Faculty of Health Sciences, Universidade de Brasilia, Brasilia, Brazil, E-mail: dorea@rudah.com.br

Claus Henn et al. (2012) addressed a “real world scenario” of exposure to multiple neurotoxic metals in their unique and interesting study. They investigated manganese–lead coexposure and its association with neurodevelopmental deficiencies in Mexican children. Their rationale was that neurodevelopmental deficiencies of both metals together could be more severe than expected based on effects of exposure to each metal alone. Indeed, they observed a synergism between manganese and lead. Given the early age of the subjects (12 and 24 months of age), I suggest that some confounders not included in their model deserve consideration in regard to this study.

Claus Henn et al. (2012) collected information on duration of breast-feeding, but it seems that in their statistical analyses, they adjusted only for sex, gestational age, hemoglobin, maternal IQ (intelligence quotient), and maternal education. Other confounders, such as thimerosal (a compound containing ethylmercury that is used as a preservative in some vaccines) and breast-feeding, may influence neurodevelopment outcomes. In countries such as Mexico, children 12–24 months of age may be immunized with thimerosal-containing vaccines (TCVs) (WHO 2011). Because of opposite effects on the central nervous system, the combination of breast-feeding and ethylmercury may influence neurodevelopmental outcomes. Kramer et al. (2008) showed that children who were exclusively breast-fed had improved cognitive development. Indeed, Kostial et al. (1978) demonstrated that infant rats fed cow’s milk diets absorbed more lead and manganese, which are associated with a higher relative retention of  mercury in the brain.

Blood levels of lead and manganese are indicators of ongoing exposure; however, ethylmercury has a short half-life and thus is unlikely to be concurrently measured in blood (Dórea et al. 2011). Nevertheless we can ascertain exposure from vaccination cards (Dórea et al. 2012; Marques et al. 2009). Following participants in the National Immunization Program of Mexico, the amount of ethylmercury from routine immunizations against hepatitis B (three doses), DTP (diphtheria, tetanus, and pertussis, three doses), and influenza can be estimated from records on vaccination cards. Additionally, during pregnancy, Mexican mothers may receive tetanus toxoid (TT) vaccines and other products, such as anti-RhoD immune globulins (given to Rh-negative mothers) that may contain thimerosal (Marques et al. 2009). These sources of prenatal and postnatal ethylmercury exposure should be considered significant sources of an additional neurotoxic coexposure—organic mercury.

Claus Henn et al. (2012) realized that information on the association of neurodevelopment and coexposure to multiple chemicals is limited; the scientific literature is even more scarce for the specific exposure to small amounts of ethylmercury derived from TCVs (Oken and Bellinger 2008), which are largely used in nonindustrialized countries. However, recent work has suggested that when studies with young children are properly adjusted for exposure to TCVs, subtle neurodevelopmental effects can be demonstrated (Dórea et al. 2012; Marques et al. 2009; Mrozek-Budzyn 2011a, 2011b). Therefore, the potential for interaction of ethylmercury, manganese, and lead provides an opportunity to expand our knowledge.

Factors related to maternal neurotoxic exposure and neurodevelopment (e.g., breast-feeding) are significant in studies of children’s exposure to ethylmercury (Marques et al. 2009). The study design used by Claus Henn et al. (2012) could provide further information on this timely issue and also provide direction for future studies of contaminants and confounders that affect neurodevelopment.
